# A Narrative Review of the Efficacy and Design of Safety Labels on Tobacco Products to Promote the Use of Safety Labels on Alcohol Products in Canada

**DOI:** 10.7759/cureus.25306

**Published:** 2022-05-24

**Authors:** Man Ting Kristina Yau, Kiana W Yau, Trana Hussaini, Eric M Yoshida

**Affiliations:** 1 Faculty of Medicine, University of Ottawa, Ottawa, CAN; 2 Faculty of Medicine, McGill University, Montreal, CAN; 3 Faculty of Pharmaceutical Sciences, University of British Columbia, Vancouver, CAN; 4 Division of Gastroenterology, Faculty of Medicine, University of British Columbia, Vancouver, CAN

**Keywords:** product labeling, health advocacy, public health, alcohol products, tobacco products

## Abstract

Alcohol is consumed by approximately three-quarters of Canadians. Alcohol causes acquired liver disease, increases the risk of cancer, has detrimental effects on mental health, and leads to adverse pregnancy outcomes. Alcohol-related morbidity and mortality are high, and urgent public health measures are warranted to prevent and control these. Tobacco safety labels have been shown in numerous studies to reduce tobacco consumption. Much can be learned from the design of tobacco safety labels in creating promising alcohol safety labels that can possibly help reduce alcohol consumption. The aim of this paper is to review the efficacy of tobacco safety labels in reducing tobacco consumption and the design of tobacco safety labels and to propose a promising design for alcohol safety labels based on our findings. English peer-reviewed papers published in western countries since 2000 were searched on PubMed and Google Scholar. Keywords and synonyms were used to search pertinent papers, which were subsequently screened by title and abstract and fully reviewed if relevant. Findings from studies comparing designs of safety labels on alcohol and tobacco products are similar. Graphics, higher emotion content, and greater size are associated with greater attention, awareness, negative emotions, intention to quit, and reduction in consumption. Mixed results are found for testimonials containing safety labels on tobacco products. It is unclear whether testimonials on alcohol safety labels reduce alcohol consumption or not. Safety labels with specific information, such as tobacco-related costs and alcohol-related cancer risks, are more effective in reducing tobacco consumption. In conclusion, preliminary alcohol safety labels show promise. Large safety labels with graphics and high emotional content appear to be most effective and may reduce alcohol consumption.

## Introduction and background

Alcohol remains one of the most used recreational substances in Canada, with over three-quarters of Canadians reporting consumption in the past 12 months as shown in annual data collected from 2008 to 2017 [1]. This is despite growing concern amongst physicians regarding its effects on the liver (i.e. alcoholic liver disease), increased risk of cancer, effects on mental health, effects on unborn children, etc. Although the prevalence of alcohol use in the past 12 months has remained stable among adult males, at around 80%, it has increased in females, particularly among those 25 years of age or older [1]. Additionally, the prevalence of alcohol use in the past 12 months continues to be the highest, at above 80% among young adults between 20 and 24 years of age [1]. Even though the legal drinking age is 18 or 19 years depending on the province or territory, nearly 50% of children between grades seven and twelve consumed alcohol in a year from 2006 to 2019, as reported in annual data from the Canadian Tobacco, Alcohol, and Drugs Survey and the Canadian Alcohol and Drug Use Monitoring Survey [1-2]. According to the low-risk drinking guidelines, the acute-risk maximum limit is three drinks for women and four drinks for men on one occasion [1]. The chronic-risk maximum limit is 10 drinks in one week and two drinks a day on most days for women and 15 drinks in one week and four drinks a day on most days for men [1]. Nineteen percent to 21% and 13%-15% of Canadians drank above acute-risk and chronic-risk maximum limits, respectively, as outlined in the low-risk alcohol drinking guidelines between 2008 and 2017 [1]. Among females, the proportion of exceeding acute-risk and chronic-risk maximum limits increased from 10.1% to 13.0% and from 16.0% to 19.2%, respectively, during this time [1].

In addition to its well-recognized detrimental effects on long-term health within the medical community, it is important to also note that alcohol use comes with many other acute risks such as decreased attention, decreased concentration, drowsiness, motor vehicle accidents, decreased memory or loss of memory, aggressive behavior, violence, respiratory arrest, and death [3-6]. If consumed on a long-term basis, it can increase the risk of not only liver diseases, but also hypertension, stroke, cancer, alcohol use disorder, depression, anxiety, and more [3-6]. In 2017, 249 per 100,000 hospitalizations were due to alcohol use, which is similar to the rate for heart attacks [7]. Between 2019 and 2020, alcohol-related mortality increased from 1.6 to 2.3 deaths per 100,000 individuals among those between 0 and 44 years of age and from 15.0 to 17.7 deaths per 100,000 individuals among those between 45 and 64 years of age [8]. In 2017, alcohol use contributed to approximately $5.43 billion in healthcare costs, $6.74 billion in lost productivity costs, $2.79 billion in criminal justice costs, and $1.66 billion in other direct costs in Canada [9]. In other words, the financial burden of alcohol on Canadian society appears to be greater than $16.5 billion annually [9].

From a public health perspective, a lot more needs to be done to protect Canadians from the negative health effects of alcohol use. Safety labels are currently mandated on tobacco products in Canada [10]. Over the past few decades, they have shown to be effective in reducing tobacco consumption around the world [10]. However, safety labels for alcohol products are not yet required in Canada [11]. It is suggested that personality (e.g. anxiety) and behavioral mechanisms (e.g. substance use reinforcement and conditioning) are similar for both alcohol and tobacco use [12]. Additionally, concurrent alcohol and tobacco use are prevalent, and alcohol and tobacco have reciprocal effects on increasing craving and consumption [13-14]. As such, given the successes seen in safety labels for tobacco products, we aim to review the efficacy and design of safety labels on tobacco products to guide the development and creation of effective safety labels on alcohol products in Canada.

## Review

Method

We searched PubMed and Google Scholar for studies in English that were published in peer-reviewed journals since 2000. The search was last updated on March 24, 2022. Year 2000 was chosen because that was when Canada first required graphic safety labels on tobacco products [15]. We screened for Canadian publications and for publications from the United States, Australia, and Europe because these countries are most similar in terms of demographics to Canada. We used search terms and their synonyms as keywords in our search strategy, including “safety labels”, “efficacy”, “design”, “tobacco”, and “alcohol”. Search terms were used in isolation and in combination. Titles and abstracts of retrieved records were screened for relevant publications. A full-text review of the relevant records was undertaken to select appropriate papers for inclusion in the current review. Reference lists of studies were reviewed for additional papers. Key findings from included studies on the designs of safety labels on tobacco products were summarized in a table. This included publication year, location of study, study design, and key findings (cognitive and behavioral changes after exposure to different safety label designs).

Efficacy of safety labels on tobacco products

The efficacy of safety labels on tobacco products has been shown in many studies. In Canada, safety labels on tobacco products must meet several requirements [16]. They must cover 75% of the front and back sides of tobacco packages, be enhanced with colors and graphics, and contain a toll-free quitline number, a web link to smoking cessation resources and services, and toxic emission statements [10,16]. Studies conducted in Canada showed that safety labels are effective at decreasing smoking rates, decreasing smoking initiation, and increasing quit attempts [17-18]. Safety labels decreased the odds of smoking initiation (OR=0.875) among a representative sample population from the Canadian National Population Health Survey [18]. In a telephonic survey, safety labels were also found to be effective in reducing the amount of tobacco consumption in approximately 20% of the study population, with the effect being the greatest among those who reported greater negative emotional responses to the safety labels [19]. Among a group of smokers who read graphic safety labels, reflected on the content, and discussed the content with others, the likelihood of attempting smoking cessation, successfully quitting smoking, and reducing tobacco consumption was greater (OR=1.07) three months after being exposed to graphic safety labels [20]. In another telephonic survey of over 9,000 participants from Canada, the United States, the United Kingdom, and Australia, participants from countries with government-mandated safety labels, including Canada, were more informed about tobacco-related health risks [21]. For example, safety labels in Canada contained information about impotence being a health risk of smoking, and participants from Canada were 2.68 times as likely as other countries to agree that smoking causes impotence [21]. The survey also noted that participants who noticed safety labels on tobacco products were more aware that smoking causes lung cancer in smokers, lung cancer in non-smokers from second-hand smoking, heart disease, stroke, and impotence [21]. Safety labels on tobacco products have also been shown to be effective in other countries. Surveys in the United States and Mexico found that safety labels increased awareness of health risks that are less known to the public, including but not limited to gangrene, impotence, and stroke [22]. Another study in the United States simulated a convenience store setting and found that graphic safety labels were effective in reducing tobacco sales from a probability of 0.71 to 0.51 among participants with low nicotine dependence [23].

Design of safety labels on tobacco products

The design of safety labels on tobacco products has been found to be critical in influencing its efficacy on smoking behavior. Design elements to consider include the size of graphic labels, the presence of graphics, high emotional content, testimonials, and specific outcomes of tobacco use (e.g. financial costs, health risks from secondhand smoking). Table 1 shows the key findings from studies assessing cognitive and behavioral changes resulting from exposure to different tobacco safety label designs.

**Table 1:** Key findings from studies assessing cognitive and behavioral changes resulting from exposure to different tobacco safety label designs

Authors	Locations of study	Study design	Key findings
Evans A. et al (2015) [24]	USA	Randomized clinical trial (n=293)	Graphic safety labels were associated with greater negative emotional responses, the credibility of safety label information, perception of tobacco-related health risks, intention to quit smoking, recall of safety label content, and knowledge of health risks than text-only safety labels. Detailed text on graphic safety labels lowered the credibility of safety label information.
Strong D. et al (2021) [25]	USA	Randomized clinical trial (n=357)	Graphic safety labels were associated with a greater negative perception of tobacco consumption, perception of tobacco health concerns, and intention to quit smoking than standard US safety labels. No significant differences were observed in smoking behavior and period of tobacco abstinence per week among those exposed to no safety labels, graphic safety labels, and standard US safety labels. Results were similar between no safety labels and standard US safety labels.
Romer D. et al (2018) [26]	USA	Randomized clinical trial (n=244)	Graphic safety labels were associated with greater negative emotional responses, lower satisfaction from tobacco consumption, and lower quantity of tobacco consumption than text-only safety labels.
Brennan E. et al (2017) [27]	Australia	Randomized clinical trial (n=924)	All interventions with graphic safety labels were associated with greater negative emotional responses, intention to quit smoking, and avoidance of safety label exposure than non-testimonial text-only safety labels. Non-testimonial graphic safety labels and graphic and textual testimonial graphic safety labels were associated with greater intention to quit. Graphic only testimonial safety labels were associated with greater quitting activity. No significant differences were found between safety labels with non-testimonial graphics, graphic-only testimonials, and graphic and textual testimonials.
Sidhu A. et al (2021) [28]	USA	Randomized clinical trial (n=361)	Graphic safety labels were found to be easy to remember, comprehensible, informative, relevant, interesting, and moderately shocking. Graphic safety labels increased perceptions of benefits associated with quitting smoking. Graphic safety labels and text-only safety labels increased attitudes towards smoking cessation and increased intention to quit. Changes observed with graphic safety labels were positively associated with the above-mentioned attributes given to graphic safety labels. However, this association was not observed for text-only safety labels.
Agaku I. et al (2015) [29]	European Union	Survey (n=26,566)	Graphic safety labels were associated with greater odds of attempting to quit and reducing the quantity of tobacco consumption than text-only safety labels. Countries that introduced graphic safety labels were associated with greater public support for plain packaging than countries that have not introduced graphic safety labels.
Macy J. et al (2016) [30]	USA	Prospective cohort study (n=2192)	Among young adults, graphic safety labels with text were associated with greater negative implicit attitudes than the U.S. Surgeon General’s text warnings and text-only safety labels. Graphic safety labels with text were also associated with greater negative explicit attitudes than U.S. Surgeon General’s text warnings.
Margalhos P. et al (2019) [31]	Portugal	Cross-sectional study (n=413)	Among adolescents, graphic safety labels were associated with greater negative emotional responses than text-only safety labels, with smokers reporting greater unpleasantness than non-smokers. Graphic safety labels were associated with a lower perception of safety label effectiveness in smokers than non-smokers.
Cantrell J. et al (2013) [32]	USA	Web-based experimental study (n=3,371)	Graphic safety labels were associated with greater emotional responses, noticeability, perception of the impact of safety labels, credibility, and intention to quit. No differences were found between race, ethnicity, level of education, and income level.
Peters E. et al (2019) [33]	USA	Randomized control study (n=1,932)	Although low-emotion graphic safety labels were associated with the greatest immediate recall, they were also associated with the greatest decrease in recall with time. High emotion graphic safety labels were associated with greater six-week recall than low-emotion graphic safety labels and greater recall was associated with a greater perception of tobacco-related health risks and intention to quit. No differences were found between high emotion graphic safety labels and text-only safety labels for a six-week recall. High emotion graphic safety labels were associated with a greater perception of tobacco-related health risks and greater intention to quit than text-only safety labels.
Droulers O. et al (2017) [34]	France	Within-subjects experiment (n=48)	High emotion graphic safety labels were associated with greater negative emotional responses and intention to quit than moderate emotion graphic safety labels. Larger sizes and plain packaging were also more likely to elicit behavioral changes toward smoking.
Berg C. et al (2012) [35]	USA	Online survey (n=24,055)	Among young adults, high emotion graphic safety labels were associated with a greater intention to quit smoking and lower smoking initiation than testimonial graphic safety labels and standard graphic safety labels.
Mead E. et al (2016) [36]	USA	Qualitative study (n=25)	Among low-income smokers, high emotion graphic safety labels were associated with a greater perception of and susceptibility to tobacco-related health risks and greater motivation to seek help to quit smoking compared to low-emotion graphic safety labels. Safety labels aimed to increase confidence in quitting smoking were associated with the greatest self-efficacy in quitting.
Kowitt S. et al (2017) [37]	USA	National survey (n=5,014)	Nearly three-quarters of participants (smokers and non-smokers) favored larger safety labels. Young age, female sex, racial and ethnic minorities, and non-smokers were more likely to prefer larger safety labels. Among smokers, females and smokers with greater intention to quit were more likely to prefer larger safety labels.
Bansal-Travers M. et al (2011)[38]	USA	Cross-sectional study (n=197)	Large graphic safety labels and safety labels focusing on negative health outcomes from smoking were rated as most attractive and most effective at increasing perception of tobacco-related health risks and increasing motivation to quit.
Skurka C. et al (2018) [39]	USA	Randomized control study (n=475)	Participants exposed to larger graphic safety labels viewed safety labels longer than smaller graphic safety labels. No difference was found between larger and smaller graphic safety labels in terms of negative emotional response and perception of tobacco-related health risks. Graphic safety labels were associated with greater negative emotional response and perception of tobacco-related health risks than no graphic safety labels. Large graphic safety labels were associated with greater intention to quit than no graphic safety labels. Among youth, no difference was found between large and small graphic safety labels in terms of susceptibility to smoking.
Brennan E. et al (2019) [40]	USA	Randomized control study (n=1255)	Testimonial graphic safety labels were associated with greater negative emotional responses, intention to quit, and quit attempts than text-only safety labels. Personal identifiers and explanatory statements decreased the effectiveness of safety labels.
Drovandi A. et al (2019) [41]	Canada, USA, United Kingdom, Australia	Online survey (n=678)	Safety labels containing information about the financial costs of smoking and the negative effects of second-hand smoking were given the highest ratings in terms of the ability to reduce smoking.
Mead E. et al (2015) [42]	USA	Qualitative study (n=25)	Safety labels containing information about the negative health effects of tobacco on smokers were described to be motivational. They increased perception of severity and susceptibility to tobacco-related health effects, increased negative emotional responses, and increased perception of risks to children. Safety labels containing information about the positive effects of quitting were found to be motivational and hopeful.

Graphic Safety Labels

Graphic safety labels have been shown to be more effective than text-only safety labels in numerous studies. Randomized clinical trials in the United States showed that graphic safety labels were associated with greater negative emotional responses, perceptions of tobacco-related health risks, intention to quit smoking, recall of content, and lower tobacco consumption as compared to text-only safety labels [24-27]. When graphic safety labels were rated on a five-point scale, they were found to be highly comprehensible, relevant, informative, easy to remember, and interesting, with mean ratings ranging between 3.4 and 4.0 [28]. In a prospective experimental study, graphic safety labels were associated with greater intention to quit smoking and avoidance of safety label exposure than text-only safety labels [27]. Even in smokers with low self-efficacy for smoking cessation, graphic safety labels were more effective than text-only safety labels in decreasing tobacco consumption as shown in an experimental study on American daily smokers [26]. Among countries of the European Union, smokers from countries that use graphic safety labels were 1.31 times as likely to reduce their tobacco consumption and attempt smoking cessation as compared to smokers from countries that use text-only safety labels [29].

Graphic safety labels also appear to benefit young adults, adolescents, and individuals from diverse socioeconomic and ethnic backgrounds. Among American young smokers between the ages of 18 and 25 years, those exposed to graphic safety labels with text were more likely to have negative implicit attitudes towards smoking than those exposed to text-only safety labels [30]. In a cross-sectional study conducted on American adolescent smokers and non-smokers, graphic safety labels were associated with greater negative emotional responses than text-only safety labels, with smokers reporting greater unpleasantness as compared to non-smokers [31]. In terms of socioeconomic and ethnic background, no difference was found in terms of education, income, race, and ethnicity in an online randomized experimental study in the United States [32]. Regardless of their background, participants exposed to graphic safety labels were more likely to report greater noticeability of the safety labels and greater impact of safety labels on smoking behavior than those exposed to text-only safety labels [32]. Graphic safety labels were also 1.41 times as likely to be rated as credible and 1.30 times as likely to motivate smokers to quit tobacco use than text-only safety labels [32].

High-Emotion Safety Labels

Another strategy to increase the efficacy of safety labels on tobacco products is to use high-emotion content. In a study conducted on 1,932 adult and adolescent smokers in the United States, participants exposed to high-emotion graphic safety labels had a greater six-week recall of label content than those exposed to low-emotion graphic safety labels, and greater recall was associated with greater perceptions of tobacco-related health risks and intention to quit smoking [33]. High-emotion safety labels were also reported to be more effective than moderate-emotion safety labels in eliciting negative emotional responses, increasing intention to quit smoking, and increasing intention to reduce tobacco consumption in a study in France [34].

Besides graphic safety labels, high-emotion safety labels were also found to be effective among young adults and smokers with low socioeconomic status. An online survey conducted on over 24,055 American college students found that 78.6% of the participants rated high-emotion safety labels to be effective at increasing intention to quit tobacco and preventing smoking initiation [35]. Interviews with smokers with low socioeconomic status showed that high-emotion safety labels were associated with greater perceptions of the severity of and susceptibility to tobacco-related health risks than low-emotion safety labels [36].

Large Safety Labels

The size of safety labels is another important factor when discussing the efficacy of safety labels. In a randomized experimental study of 5,014 American smokers and non-smokers, 72% of the participants supported larger safety labels [37]. Among smokers only and smokers with greater intention to quit smoking, 67.9% and 61% of participants, respectively, favored safety labels that covered 75% of the tobacco package [37]. Among smokers only, females and those with greater intention to quit were more likely to prefer larger safety labels [37]. Larger safety labels were also associated with greater attention, perception of tobacco-related health risks, and intention to quit smoking in other studies in the United States [38-39]. However, the size of safety labels did not appear to have an effect among adolescents [39].

Testimonials Containing Safety Labels

Mixed results have been shown from studies assessing the efficacy of safety labels containing testimonials. A study on American smokers looked at the efficacy of safety labels with images and/or personal details of real people [27]. They found that safety labels with both images and personal details of real people led to greater intention to quit smoking than text-only safety labels [27]. Safety labels with only images of real people increased actions taken to quit smoking as compared to text-only safety labels [27]. No differences were found when comparing graphic safety labels, safety labels with images of real people, and safety labels with images and personal details of real people [27]. In an online experiment conducted on 1,255 American smokers, results showed that testimonials containing graphic safety labels with images of real people and basic safety statements were the most effective [40]. Testimonials containing graphic safety labels were more effective than text-only safety labels in increasing negative emotional responses, empathy for the individual featured on the safety label, intention to quit smoking, intention to quit smoking to avoid safety label exposure, number of quit attempts, and requests for quitting information [40]. Empathy for the individual featured on the safety label was greater among those who were exposed to testimonials containing graphic safety labels with personal identifiers as compared to those without personal identifiers [40]. In comparison to testimonials containing graphic safety labels with non-testimonial explanatory statements and without any explanatory statements, testimonials containing graphic safety labels with testimonial explanatory statements were associated with lower rates of intention to quit smoking to avoid safety label exposure and lower rates of having attempted to quit smoking [40].

Safety Labels Focused on Financial Costs and Tobacco-Related Health Risks

Safety labels focused on specific topics have been found to be effective as well. In a multinational study with participants from Canada, the United States, Australia, and the United Kingdom, the highest ratings for the ability to reduce smoking were given to safety labels that contained information on the financial costs of smoking and the health effects of second-hand smoking [41]. A cross-sectional qualitative study on low-income American smokers found that safety labels focused on negative health outcomes for smokers and second-hand smokers were associated with greater perceptions of severity and susceptibility to the outcomes, negative emotional responses, concerns for children, and motivation to quit smoking [42]. Even smokers without children were affected by these safety labels and reported being more motivated to quit smoking for their future children [42].

Preliminary safety labels on alcohol products in Canada

To date, studies investigating the effects of preliminary safety labels for alcohol products in Canada have reported promising results. In a quasi-experimental study in Yukon on 2049 individuals, participants of the intervention group (exposed to alcohol products with safety labels containing cancer warnings, low-risk drinking guidelines, and standard drink information) were more likely than the control group (exposed to usual safety labels focused on alcohol use during pregnancy and driving and on possible health effects) to recall the label contents and to support the use of the new safety labels [43-44]. Additionally, participants of the intervention group were less likely to purchase alcohol that was of low cost and of greater strength [43]. The new safety labels also decreased alcohol sales by 6.59% in Whitehorse, Yukon, during the intervention period of nine months and continued to decrease sales by 10.29% five months post-intervention [45]. This is in comparison to the control group in which there was a 6.91% and a 9.16% increase in alcohol sales during and after the intervention period respectively [45]. It was also noted that the new safety labels attracted greater attention, encouraged more reflection and discussion about safety labels, and reduced a greater quantity of alcohol consumption than the usual safety labels [44,46]. Regarding the low-risk drinking guidelines, the intervention group was more likely to recall the contents with (aOR=10.8) and without prompting (aOR=7.0) and more likely to be informed of the guidelines (aOR=2.9) than the control group [47]. They were also more likely to know the daily (aOR=1.5) and weekly (aOR=1.4) drink limits [47]. Those in the intervention group who learned about the carcinogenic effects of alcohol were more likely to support policies on pricing [48].

Design of safety labels on alcohol products in and outside of Canada

Limited studies have been conducted on the design of safety labels on alcohol products in and outside of Canada, but results from these studies are like those found for tobacco products. A study conducted in Nova Scotia found that larger safety labels were associated with lower ratings for alcohol products than smaller safety labels, and plain packaging increased safety label recognition [49]. The same authors also showed that alcohol products with graphic safety labels were more negatively evaluated than text-only safety labels in terms of product-based and consumer-based perceptions [50]. In studies conducted in the United Kingdom, graphic safety labels elicited greater fear, awareness of drinking-related health consequences, and motivation to reduce and quit consumption [51-52]. Additionally, rates of selecting an alcoholic drink over a non-alcoholic drink were lower among those exposed to image and text safety labels (56%) or image-only safety labels (49%) as compared to those exposed to text-only safety labels (61%). A randomized controlled trial of 7,516 British drinkers looked at safety labels with different types of graphics that informed about alcohol content including pictographs, pie charts, and risk gradients [53]. While between 32.9% and 51.5% of those exposed to either of the graphic safety labels knew the low-risk drinking guidelines, only 21.5% of those in the control group exposed to text-only safety labels were aware of the guidelines [53]. The intervention group was also better at estimating how many servings they can drink before reaching low-risk drinking guidelines [53]. Graphic safety labels have also been more effective than text-only safety among German adolescents in eliciting greater negative emotional responses [54].

High-emotion safety labels have been demonstrated to be effective as seen in an experimental study in the United Kingdom that showed high-emotion safety labels being better than moderately high emotion safety labels in eliciting greater avoidance, opposition to the factual content of the labels, and motivation to reduce alcohol consumption [55]. Narratives on safety labels have been another strategy to increase efficacy and a study in the United States found that graphic safety labels containing narratives increased concerns about alcohol-related cancer risks and perception of the severity of getting alcohol-related cancer [56]. Safety labels focused on specific health effects of drinking can also increase efficacy. In a study on 5,528 British participants, safety labels that focused on bowel cancer as an outcome of alcohol consumption increased negative emotional responses and decreased the desire to consume alcohol [57]. More than 70% of participants in a national online survey in Australia agreed to the fact that safety labels that informed about alcohol-related cancer risks can increase awareness and discussion about the topic [58]. Moreover, several literature reviews provided suggestions regarding the design of alcohol safety labels. Five important information points to have on safety labels include ingredients, nutritional information, serving size and number of servings per alcohol product, a health warning, and the definition of moderate alcohol consumption [59]. Safety labels should also clearly communicate alcohol-related health risks, especially during pregnancy [60]. Large and colorful safety labels, safety labels on plain packaging, and messages on the negative outcomes of consuming alcohol are additional strategies that can attract attention, as outlined in a rapid review in 2021 [61].

## Conclusions

Given the success of safety labels in reducing tobacco consumption in the past decades, a lot can be learned from comprehensive research that has been conducted on tobacco safety labels. Findings from research on the designs on safety labels for tobacco and alcohol products appear similar. Studies suggest that safety labels that contain graphics, involve high emotional content, and are larger in size are more effective in attracting attention, increasing awareness about health risks, improving substance use behaviors, and increasing motivation to reduce or quit consumption. The effectiveness of testimonials containing safety labels on tobacco and alcohol products remains unclear. It has also been shown that specific contents on safety labels have been effective. For tobacco products, this includes financial costs and health risks for smokers and second-hand smokers. For alcohol products, this includes alcohol-related cancer risks. Other possible design strategies that may improve the efficacy of safety labels on alcohol products include, but are not limited to, coloring, plain packaging, and messages focused on the negative outcomes of consuming alcohol.

It is important to note that like safety labels on tobacco products, regulations need to be in place for safety labels on alcohol products to make significant, population-wide effects. Safety labels cannot solve such a large-scale societal issue on their own. Other public health measures, such as educational campaigns, social media advertisements, and school-based programs, are key to improving alcohol consumption rates in Canada. As seen with tobacco products, the effects of safety labels take decades to be observed. Alcohol, like tobacco, is one of the most abused substances in Canada, and introducing safety labels on alcohol products in Canada is long overdue. It is time for public health agencies and policymakers in Canada to act on this preventable public health issue to advocate for the health of Canadians and protect Canadians, who are currently ignorant of the negative health effects of alcohol use. It is only through effective public health measures will physicians eventually see a decrease in the number of patients dying from complications of alcohol use on a large scale. It is the duty of physicians to support, work towards, and advocate for better public health policies, including mandated alcohol safety labeling.
